# Knowledge and care regarding long-term cardiovascular risk after hypertensive disorders of pregnancy and gestational diabetes

**DOI:** 10.1007/s00508-023-02313-1

**Published:** 2024-01-03

**Authors:** Birgit Pfaller, Constance Busvine, Alena Rosenauer, Andreas Schenzel, Camille Fournier, Ida Aringer, Alexander Lösch, Martin Wiesholzer, Susanne Schubert, Barbara Wichert-Schmitt

**Affiliations:** 1 Department of Internal Medicine 1, University Hospital of St. Pölten, Karl Landsteiner Institute for Nephrology, Karl Landsteiner University of Health Sciences, St. Pölten, Austria; 2 Karl Landsteiner University of Health Sciences, Dr. Karl-Dorrek-Straße 30, 3500 Krems, Austria; 3 Department of Gynecology and Obstetrics, University Hospital of St. Pölten, Karl Landsteiner University of Health Sciences, St. Pölten, Austria; 4 Department of Cardiology and Medical Intensive Care, Kepler University Hospital, Medical Faculty, Johannes Kepler University, Linz, Austria

**Keywords:** Preeclampsia, Postpartum Care, Cardiovascular disease, Fourth Trimester, Gestational Diabetes mellitus

## Abstract

**Background:**

Adverse pregnancy outcomes (APO), such as preeclampsia (PE) and gestational diabetes (GDM) are substantial risk factors for cardiovascular disease (CVD) later in life. Identifying these high-risk female individuals during pregnancy offers the possibility of preventing long-term CVD and chronic kidney disease via a structured therapeutic and surveillance plan. We aimed to evaluate the current practice of postpartum care in women after APO and the impact on the women’s awareness about their future risk for CVD.

**Methods:**

Women diagnosed with PE and GDM at the University Hospital of St. Poelten/Lilienfeld between 2015–2020 were identified and participated in a structured telephone interview about postpartum medical care and knowledge about the impact of APOs on long-term cardiovascular health.

**Results:**

Of 161 out of the 750 women contacted, 29% (*n* = 46) were diagnosed with PE and 71% (*n* = 115) with GDM. One third of all women and up to 44% of women diagnosed with PE, were unaware that APOs are related to CVD. Women diagnosed with PE were less likely to receive postpartum care information than those with GDM (30.4% vs. 49.6%, *p* = 0.027), and only one third of all women after APOs were counselled by a physician or healthcare professional. Of the women 50% received recommendations regarding lifestyle changes after delivery; significantly more women with GDM than women with PE (54% vs. 37%, *p* = 0.05). Only 14% had at least one long-term follow-up.

**Conclusion:**

This study identified a significant deficit of structured postpartum care and a lack of awareness among women after APO and their healthcare providers about the increased risk of long-term CVD.

## Introduction

Cardiovascular disease (CVD) is the leading cause of death in women worldwide [1]. Women are particularly vulnerable to CVD, as they are often underdiagnosed, less likely to receive guideline-directed treatment and underrepresented in clinical trials [2]. Women are less informed about cardiovascular risk factors and the prevention of CVD. Classical cardiovascular risk factors are well known; however, there are additional unique female CVD risk factors and pregnancy and childbirth are specific risk periods for CVD complications in a woman’s lifetime [3]. Preeclampsia/hypertensive disorders of pregnancy (PE/HDP) are diagnosed in 2–8% of pregnancies [4]. Preeclampsia can be classified as early-onset preeclampsia and late-onset preeclampsia with the diagnosis prior to and/or after 34 weeks of gestation. Women diagnosed with early-onset preeclampsia present with a more severe degree of organ involvement and are at higher risk of long-term cardiovascular disease compared to normotensive women [5]. Women diagnosed with hypertensive disorders of pregnancy have a significantly increased risk of future CVD compared to normotensive pregnancies, a 2–8-fold increased risk of chronic hypertension, 2‑fold increased risk of coronary artery disease and increased risk of stroke [5–8]. One in five women with hypertensive disorders of pregnancy will be diagnosed with hypertension within 15 years postpartum [9]. Furthermore, women with hypertensive disorders are more likely to develop type 2 diabetes [10, 11] and chronic kidney disease [12–14]. Gestational diabetes affects approximately 14% of pregnancies worldwide and increases the risk of CVD in the long term. Women with GDM have a 2-fold increased risk of cardiovascular events [15] and a lifetime risk of being diagnosed with type 2 diabetes of 50–60% [16]. Preventive programs that include counselling about health risks and lifestyle changes demonstrated a 40% risk reduction of type 2 diabetes in the general population [17]. While it is generally recommended in guidelines that women after adverse pregnancy outcomes (APO) should receive postpartum counselling due to their enhanced CVD risk profile [2, 18–22], there is a lack of care and knowledge transfer to women and primary healthcare providers after hypertensive disorders of pregnancy and GDM [23].

Even though there are well-known and established risk factors for CVD and chronic kidney disease (CKD) [6, 7, 24], these are not acknowledged by healthcare providers and women [23, 25]. Different national and international guidelines and recommendations exist regarding midterm and long-term surveillance plans after APO [26]. For example, the 2021 International Society for the Study of Hypertension in Pregnancy classification, diagnosis and management recommendations for international practice proposed that all women should be evaluated at 3–6 months postpartum to ensure that blood pressure, proteinuria, and other laboratory abnormalities have normalized. Furthermore, every year for the first 5–10 years postpartum, a yearly medical follow-up is recommended [4].

This study aimed to outline the status quo of postpartum care concerning PE and GDM. We conducted a survey to evaluate the present status of postpartum care and risk awareness of women with diagnosed gestational diabetes (GDM) or preeclampsia (PE).

## Methods

Women who gave birth between 1 January 2015 and 31 October 2020 at the University Hospital of St. Poelten/Lilienfeld and were diagnosed with PE or GDM, were identified through the hospital information system. An invitation letter to participate in our survey was sent to 750 women. The letter outlined the aim of this study and the mode of contact and provided an e‑mail address to decline participation in a nonverbal way or to specify a time to be contacted. Women were included if they were German-speaking, 18 years or older and if the diagnosis of hypertensive disorders in pregnancy or GDM was confirmed via electronic hospital records (BP). In accordance with the study period (2015–2020), the international 2013 definition by the American College of Obstetricians and Gynecologists Task Force on Hypertension in Pregnancy was applied to define PE [20]. A structured interview was conducted from June 2021 to October 2021 after women gave oral consent. As a validated German questionnaire for assessing women’s risk perception and knowledge was not available, a survey was custom designed using the publications summarized in the review “Assessing Knowledge Gaps of Women and Healthcare Providers Concerning Cardiovascular Risk After Hypertensive Disorders of Pregnancy—A Scoping Review” [23]. The interview process: trained interviewers performed (AR, CB) and included a survey on received postpartum care, counselling about cardioprotective strategies, and risk perception. The data collection sheet was based on “The Mothers Program^TM^” and related protocols [23, 27, 28]. Additional patient characteristics collected were maternal age and body mass index (BMI), gestational age at delivery, gravidity and parity, ethnicity, smoking history, presence of medical conditions in hypertension and diabetes mellitus, medication, prior gestational diabetes, prior hypertensive disorders of pregnancy and family history.

The study was approved by the Karl Landsteiner University of Health Sciences Ethics Committee of the University (1099/2020) and performed according to the Declaration of Helsinki; informed consent to participate in the study was obtained from all women. All data were analyzed using standard statistical packages (SPSS version 27 Windows, IBM Corp. Armonk, NY, USA). Data are presented as means ± standard deviation (maternal age, gestational week of delivery, gestational week of diagnosis) and categorical variables as proportions. The χ^2^-test Student’s t‑test or Fisherʼs exact test were used to compare these two groups as appropriate. Pregnant women who were diagnosed with both conditions (pre-eclampsia and gestational diabetes) were excluded. The two-sided *p* values < 0.05 were considered significant.

## Results

**Fig. 1:**
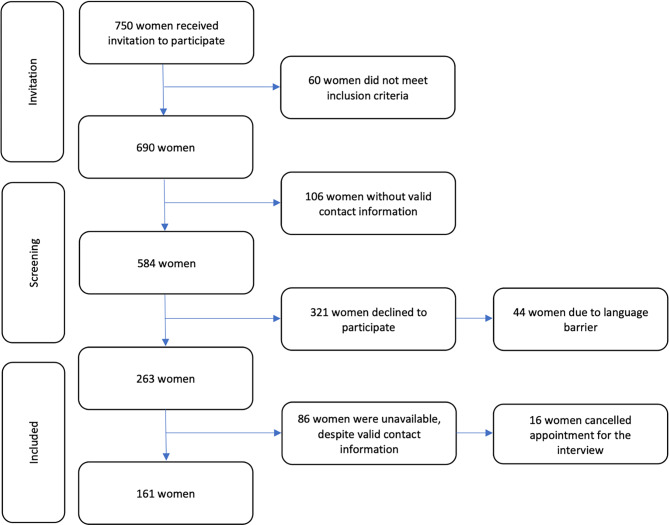
Flow chart of study participants

After the screening shown in Fig. 1, 161 women completed the telephone interview and were included in our study. In total, 46 (29%) women were diagnosed with preeclampsia (PE) and 115 (71%) women with gestational diabetes (GDM). Differences in characteristics of women with GDM compared to those with PE are shown in Table 1. Women with PE were younger at the time of delivery than women with GDM (30.0 vs. 32.8 years, *p* = 0.002). Prepregnancy mean body mass index (BMI) was 26.5 kg/m^2^, and over one quarter of women were obese (BMI > 30). The percentage of women with obesity was similar in PE and GDM women (24% vs. 27%, *p* = 0.7). Overall, 96% (*n* = 154) of women were European. Women diagnosed with GDM tended to be regularly physically active compared to women with PE (68% vs. 54%, *p* = 0.12). Almost one half of the women (47.5%) reported ever using tobacco in the past, with a similar prevalence observed in both groups, and 16.1% were current smokers at the time of the survey. About 50% of women reported a positive family history of chronic hypertension, 67.4% of women with PE and 46.1% with GDM. Half of all women (49.1%) reported a family history of type 2 diabetes mellitus, 55.7% of women with GDM compared to 32.6% of women with PE (*p* = 0.008). Of the women with PE 28 (61%) had early onset preeclampsia. Gestational age at delivery was lower in the PE group (35.4 vs. 38.8 weeks, *p* < 0.001), 16 of the 115 (13.9%) women diagnosed with GDM had a past medical history of GDM, and 17.4% (*n* = 8) of women diagnosed with PE had already been diagnosed with chronic hypertension and suffered from superimposed PE. Women with PE had a significantly more extended hospital stay than women diagnosed with GDM (37.0% vs. 1.7%, *p* < 0.001) and 3 women diagnosed with PE were admitted to the intensive care unit (ICU) after giving birth. No ICU admission was reported during pregnancy.

**Table 1:** Baseline characteristics of women diagnosed with PE or GDM

	Total (*n* = 161), mean (SD) or *n* (%)	GDM (*n* = 115), mean (SD) or *n* (%)	PE (*n* = 46), mean (SD) or *n* (%)	*p*-value
Maternal age before delivery, years, mean ± SD	32.0 (± 5.1)	32.8 (± 5.0)	30.0 (± 4.9)	0.002
Maternal age at survey, years, mean ± SD	35.2 (± 5.3)	36.0 (± 5.3)	33.4 (± 5.0)	0.006
Prepregnancy body mass index (BMI) kg/m^2^, mean ± SD (*n* = 160)	26.5 (± 6.3)	26.7 (± 6.8)	25.9 (± 4.9)	0.48
Prepregnancy BMI > 30, *n* (%) (*n* = 160)	42 (26.1)	31 (27.0)	11 (24)	0.7
*Smoker, n (%)*				
Ever used tobacco (*n* = 160)	76 (47.5)	52 (45.6)	24 (52.2)	0.25
At survey	26 (16.1)	21 (18.3)	5 (10.9)	0.45
Regular physical activity, *n* (%)	103 (64.0)	78 (67.8)	25 (54.3)	0.12
Gestational age at delivery, weeks, mean ± SD (*n* = 160)	37.8 (± 3.5)	38.8 (± 2.8)	35.4 (± 3.8)	< 0.001
Twins or multiples, *n* (%)	9 (5.6)	5 (4.3)	4 (8.7)	0.28
Previous chronic hypertension, Diabetes mellitus, *n* (%)	15 (9.3)	4 (3.5)	11 (23.9)	< 0.001
Chronic hypertension, *n* (%)	11 (6.8)	3 (2.6)	8 (17.4)	0.002
Prior preeclampsia, *n* (%)	6 (3.7)	4 (3.5)	2 (4.3)	1.0
Prior gestational diabetes mellitus, *n* (%)	16 (9.9)	16 (13.9)	0 (0.0)	0.006

*PE* Preeclampsia, *GDM* Gestational Diabetes Mellitus

Values are represented as mean ± SD or *n* (%)

### Postpartum information regarding long-term CVD risk

Women diagnosed with GDM were more likely to receive postpartum care information than women diagnosed with PE (49.6% vs. 30.4%, *p* = 0.027) (Table 2). Counselling regarding the increased long-term risk of developing cardiovascular disease later in life was provided to one third of women (GDM vs. PE; 27% vs. 28%, *p* = 0.87). Of the women 19 (11.8%) received information about the increased CVD risk from their obstetrician, while 27 (16.8%) were counselled by their general practitioner or internal medicine specialist. Women diagnosed with PE were significantly less likely to be informed about their increased risk of CVD by their general practitioner or internist than those diagnosed with GDM (4.3% vs. 21.7%, *p* = 0.008). Information highlighting the necessity of long-term follow-up with a healthcare provider was given to one fifth of women (23% GDM and 20% PE, *p* = 0.59). Overall, 40% of women scheduled a visit with their primary healthcare provider/general practitioner in the first months after delivery. In total, 14% attended at least one long-term follow-up after their adverse pregnancy complications.

**Table 2:** Postpartum care recommendations information received of women diagnosed with PE or GDM in Lower Austria

	Total (*n* = 161) mean (SD) or *n* (%)	GDM (*n* = 115) mean (SD) or *n* (%)	PE (*n* = 46) mean (SD) or *n* (%)	*p*-value
Information regarding postpartum care after high-risk pregnancy received *n* (%)	71 (44.1)	57 (49.6)	14 (30.4)	0.027
Information provided regarding increased risk of CVD *n* (%)	44 (27.3)	31 (27.0)	13 (28.3)	0.87
Information received regarding long-term follow-up due to increased risk of CVD *n* (%)	36 (22.4)	27 (23.5)	9 (19.6)	0.59
Recommendation received regarding long-term follow-up and at least 1 follow-up visit attended *n* (%)	23 (14.3)	15 (13.0)	8 (17.4)	0.34
Regular visits general practitioner in the first months after delivery *n* (%)	64 (39.8)	45 (39.1)	19 (41.3)	0.9
Recommendation of lifestyle change received *n* (%)	79 (49.1)	62 (53.9)	17 (37.0)	0.05
Recommendation regarding healthy diet received *n* (%)	91 (56.5)	76 (66.1)	15 (32.6)	< 0.001
Recommendation regarding healthy weight *n* (%)	71 (44.1)	54 (47.0)	17 (37.0)	0.25
Recommendation of regular physical exercise received (150 min/per week) *n* (%)	71 (44.1)	55 (47.8)	16 (34.8)	0.13
Postpartum adaption disorder or postpartum depression assessed *n* (%)	9 (5.6)	7 (6)	2 (4)	1.00
General recommendation for the next pregnancy received *n* (%)	48 (29.8)	34 (29.6)	14 (30.4)	0.91
Information regarding recurrence in next pregnancy *n* (%)	110 (68.3)	83 (72.2)	27 (58.7)	0.097

*PE* Preeclampsia, *GDM* Gestational Diabetes Mellitus, *CVD* cardiovascular disease

Values are represented as mean ± SD or *n* (%)

#### Recommendations regarding lifestyle and mental health advice

Of all women 50% received recommendations for a healthier lifestyle after delivery. This was significantly more likely to occur in women with GDM than in women with PE (54% vs. 37%, *p* = 0.05). In detail, women with GDM had been more commonly advised to change their diet (66% vs. 33% in women with PE, *p* < 0.001), informed about a healthy weight (47% vs. 37% in women with PE, *p* = 0.25) and regarding regular physical activity (48% vs. 35% in women with PE, *p* = 0.13). Women with adverse pregnancy outcomes are at risk of postpartum depression. Still, only 6% of women had been assessed for mood disorders via questionnaires or consultation, and 2 had been diagnosed with postpartum depression. Out of 161 women 11 were on antidepressants.

#### Recommendations on management, risks, and lifestyle changes in future pregnancies

One third, equally distributed between both groups, received recommendations regarding a subsequent pregnancy. Information on the possible recurrence of PE was provided to 59% of women with PE and the recurrence of GDM to 72% of women with GDM. In total, 43% of women with PE received information regarding preventive measurements of PE in the case of a future pregnancy.

#### Risk awareness and knowledge about long-term CVD risk

One third of women were unaware that adverse pregnancy outcomes are related to CVD (Table 3). In women diagnosed with PE, 44% were unaware of any increased risk of CVD in the long term. One half of women diagnosed with PE knew about the increased risk of being diagnosed with chronic hypertension later in life. In contrast, 77.4% of women diagnosed with GDM were aware of the long-term risk of diabetes, and 47.8% knew about the long-term overall increased risk for CVD.

**Table 3:** Risk awareness regarding long-term risks after diagnosis of preeclampsia or gestational diabetes

	GDM(*n* = 115)	PE(*n* = 46)	*p*-value
Can you state the long-term risk following your pregnancy complication?			
Type 2 diabetes mellitus, *n* (%)	89 (77.4)	2 (4.3)	< 0.001
Hypertension, *n* (%)	46 (40.0)	23 (50.0)	0.25
Cardiovascular disease^a^, *n* (%)	55 (47.8)	20 (43.5)	0.62
Stroke, *n* (%)	33 (28.7)	12 (26.1)	0.74
Coronary heart disease, *n* (%)	50 (43.5)	17 (37.0)	0.45
Myocardial infarction, *n* (%)	41 (35.7)	16 (34.8)	0.92
No long-term risk, *n* (%)	22 (19.1)	20 (43.5)	0.001

*PE* Preeclampsia, *GDM* Gestational Diabetes Mellitus, *CVD* cardiovascular disease

^a^ CVD includes participants that stated stroke, CHD or MI as a long-term risk

## Discussion

This study evaluated the current practice of postpartum care and healthcare provider counselling of women diagnosed with preeclampsia (PE) and gestational diabetes mellitus (GDM) and the women’s knowledge and awareness about long-term risks for CVD. The peripartum period represents the perfect window of opportunity for assessing cardiovascular risk factors, counselling regarding lifestyle changes and smoking cessation in a cohort of high-risk individuals at a young age. The high prevalence of obesity and smoking in our study further highlights the need for structured postpartum care as a chance for the primary prevention of cardiovascular disease in women.

### Postpartum care and long-term CVD risks

Overall, 44.1% of participants had been informed regarding postpartum care following a high-risk pregnancy. Only half of the women were satisfied with the information given and only 39.8% of women visited their general practitioner regularly within the first months after childbirth. This indicates that women with high-risk pregnancies may not receive adequate treatment in the postpartum period, enhancing the risk for immediate complications such as stroke, cardiovascular, obstetric and renal complications in women diagnosed with PE [29]. Of note, around half of the fatal maternal cardiovascular events related to pregnancy occur in the postpartum period [30]. This fact underlines the urgent need for healthcare provider education and improving postpartum care structures.

Several studies reported that information regarding the increased long-term CVD risk was given to only a minority, around one quarter of women with adverse pregnancy outcomes such as PE and GDM. [31–33]. In our study, counselling regarding long-term CVD risk was provided to about one third of women diagnosed with GDM or PE. Information regarding the importance of regular long-term follow-up consultations with a healthcare provider was only given to one fifth of women. Accordingly, only 14.3% attended at least one long-term follow-up visit after their adverse pregnancy complication. This indicates that women with high-risk pregnancies lack the required information to adhere to and participate in long-term care plans with their GP, missing the chance of early detection of new onset chronic hypertension and type 2 diabetes and mitigation of their long-term risk for CVD by timely initiation of adequate treatment. [26, 34].

### Recommendations for lifestyle changes

Notably, many risk factors for preeclampsia and GDM overlap with traditional CVD risk factors [34]; nevertheless, only 50% of all women received lifestyle recommendations after delivery. In our study, women diagnosed with GDM more frequently received lifestyle recommendations than women with PE and tended to be more frequently active than women with PE. GDM pregnancy and postpartum care are more standardized in Austria than care of women with hypertensive disorders of pregnancy, and this might lead to long-term consequences for being physically active and maintaining a healthier lifestyle in women with GDM. Educating young women seems particularly efficient as their motivation to stay healthy for their family should be high, and their behavior might also transmit to a healthier lifestyle for their children [35]; however, randomized controlled trials are needed to provide information regarding the effectiveness of interventions to reduce CVD risk factors and CVD outcomes in women [36, 37]. As depression has been recognized as an important risk factor for CVD, particularly in women, healthcare providers pay attention to postpartum mental health issues and offer adequate help and treatment [38].

#### Next pregnancy

This study matches Bijl et al.’s findings where approximately two thirds of women with PE stated being informed about the recurrence risk of PE [32]. Less than one half of women diagnosed with PE (43%) received information about measures to reduce the risk of PE in future pregnancies. Given that the recurrence of PE in a subsequent pregnancy can be deleterious for the women and the fetus, healthcare providers must address this crucial aspect during counselling and provide information regarding preeclampsia prevention and low-dose aspirin for women at risk [4].

#### Limitations

In this study, we used a custom-created survey as a validated German questionnaire to assess women’s risk perception, and knowledge was unavailable. We verified pregnancy-related diagnosis; however, other conditions have been self-reported. We could not include women with language barriers, and these women might have different awareness and understanding. Women with mental health diseases typically underreport their diagnosis, and therefore, the rates may be underreported in our study but not likely overreported. The main limitation is the recall bias of the information received, a pilot study between different periods between the event and interview and the perceived message would be beneficial.

## Conclusion

Our findings show that the current practice of postpartum care of women diagnosed with gestational diabetes or preeclampsia is insufficient to inform women after adverse pregnancy outcomes about the future risks of CVD, guide them to address their risk factors and maintain regular surveillance. We strongly encourage that more attention be paid to healthcare provider education and the implementation of structured postpartum care plans to provide proactive counselling on cardiovascular risk, risk factors and primary prevention of CVD to women diagnosed with gestational diabetes and hypertensive disorders of pregnancy in order to improve their long-term cardiovascular health. Postpartum care plans should be recommended at 3–6 months postpartum, and depending on the diagnosis, follow-up appointments should be recommended with their primary healthcare providers. Hypertensive disorders of pregnancy and gestational diabetes should be considered as a screening tool for future cardiovascular disease prevention through national initiatives.

## Abbreviations

APO: Adverse pregnancy outcomes
CVD: Cardiovascular disease
GDM: Gestational diabetes
HDP: Hypertensive disorders of pregnancy
PE: Preeclampsia
